# Proprioceptive Focal Stimulation (Equistasi®) May Improve the Quality of Gait in Middle-Moderate Parkinson's Disease Patients. Double-Blind, Double-Dummy, Randomized, Crossover, Italian Multicentric Study

**DOI:** 10.3389/fneur.2019.00998

**Published:** 2019-09-18

**Authors:** Antonella Peppe, Stefano Paravati, Maria Giulia Baldassarre, Leila Bakdounes, Fabiola Spolaor, Annamaria Guiotto, Davide Pavan, Zimi Sawacha, Sonia Bottino, Daniela Clerici, Nicola Cau, Alessandro Mauro, Giovanni Albani, Micol Avenali, Giorgio Sandrini, Cristina Tassorelli, Daniele Volpe

**Affiliations:** ^1^IRCCS Santa Lucia Foundation, Rome, Italy; ^2^Parkinson Excellence Center of the Fresco Institute for Italy, Villa Margherita Clinic of Vicenza, Vicenza, Italy; ^3^Department of Information Engineering, University of Padova, Padua, Italy; ^4^Department of Neurology, Italian Auxological Institute, IRCCS, Verbania, Italy; ^5^Politecnico of Milano, Milan, Italy; ^6^University of Torino, Turin, Italy; ^7^Department of Brain and Behavioral Sciences, University of Pavia, Pavia, Italy; ^8^Department of Neurology and Neurorehabilitation, Mondino Foundation, Pavia, Italy

**Keywords:** Parkinson's, gait analysis, Equistasi®, proprioception, Focal-proprioceptive stimulation, middle-moderate

## Abstract

**Objective:** The object of the study was to evaluate the efficacy of Proprioceptive Focal Stimulation on Gait in middle—advanced Parkinson (PD) patients by a crossover, randomized, double Blind double dummy study using Equistasi®, a nano-technological device of the dimension of a plaster which generates High Frequency Vibration (FV).

**Background:** The efficacy of Gait Analysis (GA) on evaluating gait modification on Parkinson's disease (PD) Patients is already well-known. Therefore, GA was recorded in a group of PD patients using Equistasi® device and its placebo.

**Methods:** Forty PD patients on optimal therapy were enrolled in the study. Patients were randomly assigned to receive active or sham stimulation for 8 weeks and, following a wash-out period, switched to an additional 8-week period with the reverse intervention. GA was performed at baseline and at the end of both 8-weeks treatment periods Clinical state was monitored by MDUPDRS part III.

**Results:** Active stimulation induced a significant improvement in Mean Velocity (Velocity), Stride Length (SL), Stance (STA), and Double Support (DST) percentage, both in left and right stride. The ANOVA analysis using H&Y stage as a factor, showed that DST and MDUPDRS III scores improved significantly more in the more severely affected subjects.

**Conclusions:** The findings obtained in this randomized controlled study show the efficacy of mechanical focal vibration, as stimulation of the proprioceptive system, in PD and encourage further investigation. The effect of the device on more severe patients may open a new possibility to identify the most appropriate candidate for the management of gait disturbances and postural instability with FV delivered with Equistasi®.

## Introduction

Parkinson's disease (PD) is the second most frequent neurological disease. PD is a movement disorder, but it involves many different pathways of the Central Nervous System (i.e., Vestibular system or CM/PF complex pathways). Gait disorders, balance impairment, falls, and fall-related injuries have frequently reported by PD patients (1). Indeed, patients with PD demonstrate impaired ability to walk (2, 3) and to change direction (4). These symptoms are not only related to motor pathways, as they involve different circuits, including proprioception (5). The Clinical management of PD has traditionally been based on pharmacological and/or surgical therapy, yet, even with optimal medical management, PD patients experience deterioration in daily activities (6), especially in gait and balance. As disease progresses, these features worsen, treatment efficacy wanes, and gait impairment becomes increasingly disabling (4). In this case, the advanced pharmacological therapy and the Deep Brain stimulation (DBS) of subthalamic nucleus is not enough to reduce the balance impairment. Many studies using stereophotometric recording of Gait (GA) have shown gait alterations in PD patients (7) and have identified Dynamics and Kinematics gait parameters related to falls (8, 9). The aim of the present study is to verify the clinical impact of the modulation of the proprioceptive system in the gait performance of PD subjects using the Equistasi®® device. In a previous study, the action of Equistasi® in a group of PD patients related to rehabilitation training was seen; in this study, plaquettes of Equistasi® were added to pharmacological therapy and no rehabilitation training during the study was performed by patients. Equistasi® is a Class I Medical Device with European Certification registered in Italy as Postural Stabilater#231535. The fibers of Equistasi®, polymer exclusively made of nanotechnological fibers, are very sensitive to the smallest variation in temperature. These fibers release FV transforming thermal energy that they receive from the skin into mechanical energy. FV starts a few seconds after application of the device on the patient's skin (10, 11). More specifically, the primary end-point of the study is to evaluate the positive effect of Equistasi® on spatial temporal variables of GA. The secondary end-point is the evaluation of the efficacy of the device on clinical scales.

## Methods

### Study Design

This is a multicentric, randomized, double-blind crossover study vs. Placebo. Forty patients diagnosed with idiopathic PD were enrolled in 4 rehabilitation centers in Italy: S. Lucia Foundation in Rome, the Auxologic Institute of Piancavallo Verbania, the Villa Margherita Clinic in Vicenza (Fresco Parkinson Institute) and the Mondino Foundation Neurological Institute of Pavia. Each center obtained approval from the local ethics committee (protocol number CE/PROG 478/15 del 19/11/2015, 58/16, 61/16, 60/16, respectively). After screening and enrollment, patients were randomized to receive proprioceptive mechanical stimulation for 8 weeks with either Equistasi®® (12) or Placebo, in the absence of any other rehabilitative procedure. At the end of the first 8-week period and following a 4-week wash-out period, the patients were switched to the second 8-week period of reverse stimulation. Written informed consent was obtained by all the participants.

### Subjects

All patients in this study suffered from a rigid akinetic form of bilateral Idiopathic Parkinson's Disease [Hoehn and Yahr (13): 2–3] according to current criteria (14). No specific subtypes of PD were involved in the study, patients had to be autonomous in walking and in performing the required tasks, therefore patients who presented significant freezing of gait or those who showed important balance disorders were excluded. Disease duration was >5 years and all subjects showed a good response to anti-Parkinsonian therapy and had been on stable treatment regimens for at least 3 months. During this study pharmacological treatment was kept unmodified. The exclusion criteria were: presence of co-morbidities that might prevent safe mobility (including clinically evident neuropathy and important medical conditions such as malignant tumors), severe dysautonomia with marked hypotension, major depression, dementia, pregnancy, cardiac pace-maker, DBS, or other conditions affecting postural stability as well as poor visual acuity or vestibular dysfunction. In addition, patients had to have a MMSE score > 24 (15) (Table 1).

**Table 1:** Clinical characteristics of the PD group (Mean and Standard deviation).

	**Mean**	**± SD**
Sex (M/F)	26/14	
R/L/bilateral	24/15/1	
AGE at onset symptoms (years)	60.27	9.9
PD duration (years)	8.347	3.6
Age at onset antiparkinsonian therapy	61.36	9.9
Therapy duration (years)	7.39	3.9
Antiparkinsonian Therapy dose (mg)	743.3	293
H&Y	2.45	0.50
MMSE	27.11	1.88
BMI	25.69	3.6

*R, right onset; L, Left onset; PD duration, years of Parkinson disease duration; Therapy Duration, years of antiparkinsonian treatment duration; Antiparkinsonian Therapy dose: LD and LEDD, Levodopa Equivalent Daily Dose; H&Y, Hoehn & Yahr stage; MMSE, Mini-Mental State Examination; BMI, Body Mass Index*.

### Randomization and Blindness

A series of random numbers without repetition (from 0 to 300) were created. Each number was alternately associated with a kit containing three active devices or a kit containing three placebo devices. Each pair of kits (one placebo and one active) were put into a box and sent to the researcher. Each single box was associated with a patient, who randomly chose one of the two kits as the first therapy and the other one as the second. In this way, both the patient and the researcher were both blind to the device type.

### Intervention

**Figure 1:**
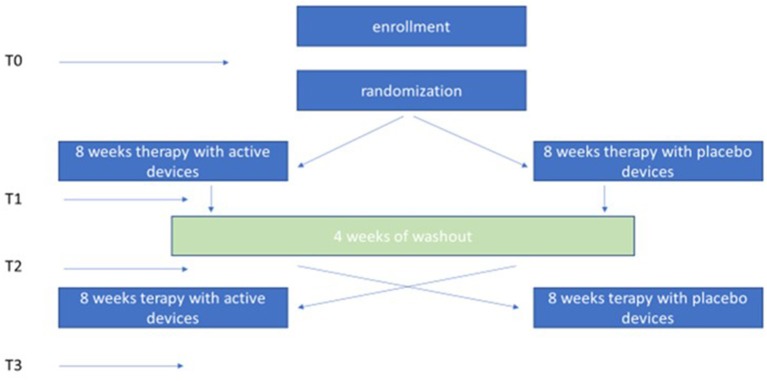
Study design.

Once the informed consent had been obtained for participation in the study, the patients were recruited (T0) and evaluated for the primary and secondary endpoints. Thereafter, patients chose one of the two kits within the box assigned. The 3 plaques contained in the box were positioned on the skin as follow: one over the 7th cervical vertebra and on each soleus muscle tendons, according to literature data (12). The device was worn 6 days/week for 1 h during the first week. The wearing time increased by 1 h per week until the 4th week, when the device was worn 4 h/day. The wearing time was stable for the subsequent 4 weeks during which the device was worn 5 days/week. At the end of the first 8-week period, patients were reevaluated for primary and secondary end points (T1) and subsequently entered a 4-week wash-out period. Once the wash-out period was completed, patients were re-evaluated for primary and secondary endpoints (T2) and then received the other kit assigned to them for continuing for the crossover treatment. At the end of the second 8-week treatment the patients were re-evaluated for primary and secondary endpoints (T3) (Figure 1). Telephone contacts with caregivers and patients allowed to monitor the consistency of the study.

### Outcome Measures

We assessed outcomes at four time points:

– Baseline (T0) before enrolling;
– second assessment (T1) at the end of the first 8-week treatment period;
– third assessment (T2) at the beginning of the second 8-week treatment, i.e., at the end of the 4-week wash-out period;
– fourth assessment (T3) at the end of the second 8-week treatment period.

Evaluation has performed always in the morning, in ON state 60' after the first dose of Levodopa.

#### Instrumental Assessment

Gait Analysis was performed with 6 cameras with stereophotogrammetric system (BTS Smart system) according to the modified Davis Protocol (16) in all centers. The 6 video cameras were positioned along a 10 m walkway with a sampling rate of 50 Hz, a 640 pixel of resolution, and a 6 mm lens. After three-dimensional (3D) calibration, the spatial accuracy of the system was <0.5 mm. Spatial temporal variables as velocity (Velocity), stride length (Stride Length), Stride Phase Percentage of the Stance (Stance), and Double Support (DST) were taken into account [For more details see (8)].

#### Clinical Assessment

Motor impairment was evaluated using the parts III (Motor Examination) of the Movement Disorders Unified Parkinson's Disease Rating Scale (17) and Items 3.10, 3.11, 3.12, 3.13 were separately considered for underlying data on gait, freezing of gait, postural, and postural instability of PD patients. Other data collected at baseline included age, gender, body mass index (BMI), disease duration, Hoehn & Yahr stage, anti-Parkinsonian treatment expressed as levodopa-equivalent daily dose (18) and cognitive status assessed with the MMSE. All adverse events such as injuries were verified and recorded during the study (Table 1).

### Outcome Measures

Since the mean Velocity has been used as a reliable parameter in GA and in previous study using FV reports for mean Velocity variable, a Standard Deviation of 15.7 cm/s without and 17.3 with vibration (19); we expected an effect around 10 cm/s and with this expectation we calculated the power of the study. With a two-tailed, type I error of 0.05 and a power of 80%, the estimated sample size was 40 patients. The calculation of this number was obtained in accordance with Cohen (20) and Norman et al. (21).

### Statistical Analysis

We verified the normality of the distribution of the variables with the Shapiro-Wilk test and the effect of the treatments (Placebo—Equistasi®) through the “PRE” and “POST” time points, with ANOVA for repeated measurements after grouping the times for each treatment and for each patient. We used ANOVA for repeated one-way measurements, to evaluate the significance of the H&Y factor, on the efficacy of treatments. For variables that did not meet the normal assumptions we used non-parametric tests and the necessary information on the adequacy of the *p*-value estimates: we verified them with the confidence interval (22) and used the Monte Carlo method (MC) (23). The potential “Carry-Over” effect was evaluated between T0 and T2. For all parametric variables, the Student *t*-test for paired samples was used. Mean and Standard Deviation expressed all values and IBM SPSS Statistics ver. 20.0 we used for all statistical analysis. All tests were bilateral with a level of significance set at *P* ≤ 0.05.

## Results

Complete data sets from 40 patients were obtained. No carry-over effect was observed comparing the GA parameters and the score of clinical scales recorded at T0 and T2 (Table 2). No major adverse events during the study period were reported.

**Table 2:** Difference between the means of the variables at the beginning of the two treatments.

	**T0**	**T2**	*p* value^*^
	**Mean (±SD)**	**Mean (±SD)**	
UPDRS III TOTAL SCORE	33.2 (13.1)	30.2 (12.4)	0.31
UPDRS III SCALE ITEM 10	1.51 (0.9)	1.51 (0.8)	0.91
UPDRS III SCALE ITEM 11	0.64 (0.9)	0.53 (0.9)	0.65
UPDRS III SCALE ITEM 12	1.33 (1.0)	1.34 (0.9)	0.90
UPDRS III SCALE ITEM 13	1.58 (1.1)	1.53 (1.1)	0.78
Mean Velocity (m/s)	0.70 (0.23)	0.74 (0.21)	0.34
STRIDE LENGTH RIGHT (m)	0.85 (0.23)	0.88 (0.24)	0.44
STRIDE LENGTH LEFT (m)	0.85 (0.21)	0.87 (0.22)	0.52
STANCE (% CYCLE) RIGHT	64.4 (3.22)	63.7 (4.1)	0.62
STANCE (% CYCLE) LEFT	64.3 (3.11)	64.2 (3.87)	0.82
DST (% CYCLE) RIGHT	14.9 (3.3)	14.4 (4.1)	0.80
DST (% CYCLE) LEFT	14.8 (3.8)	14.1 (4.2)	0.68

* *t- Student test for dependent sample*.

### Effects of Two Treatments

After the opening of the randomization the active treatment was separated from the placebo and we verified the efficacy of the two treatments in the kinematics and clinics parameters.

#### Kinematic Parameters

In the active treatment, we observed a significant improvement in Mean Velocity from 0.70 to 0.75 m/s *p* = 0.006; a significant increase in the length of the Stride for both right and left from 0.85 to 0.91 m *p* = 0.003 and from 0.84 to 0.89 m *p* = 0.005, respectively. Significant reduction in the right and left Stance percentage from 64.8 to 63.6%. *p* = 0.026 and from 64.7 to 63.7% *p* = 0.04, respectively, furthermore a reduction of the right and left DST percentage from 14.2 to 13.3% *p* = 0.036 and from 14.7 to 13.8% *p* = 0.007, respectively. No significant differences were observed on the kinematic variables of the gait in Placebo treatment (Table 3).

**Table 3:** Effectiveness of two treatments.

	**Active device**			**Placebo device**		
	**Pre**	**Post**	***p* value**	**Pre**	**Post**	***p* value**
	**Mean (±SD)**	**Mean (±SD)**		**Mean (±SD)**	**Mean (±SD)**	
Velocity (m/s)^*^	0.70 (0.25)	0.75 (0.23)	0.006	0.73 (0.22)	0.72 (0.25)	0.459
Stride length R (m)^*^	0.85 (0.25)	0.91 (0.24)	0.003	0.87 (0.21)	0.86 (0.2)	0.123
Stride length L (m)^*^	0.84 (0.16)	0.89 (0.25)	0.005	0.87 (0.25)	0.86 (0.26)	0.215
Stance R (%)^*^	64.8 (3.4)	63.6 (3.6)	0.026	63.6 (4.3)	64.6 (6.7)	0.352
Stance L (%)^*^	64.7 (2.9)	63.7 (4.1)	0.040	63.9 (4.1)	64.3 (8.1)	0.435
DST R (%)^*^	14.2(3.9)	13.3 (3.5)	0.036	14.9 (4.0)	14.2 (3.9)	0.472
DST L (%)^*^	14.7 (3.7)	13.8 (3.1)	0.007	14.8 (4.7)	14.4 (4.1)	0.543
UPDRS III TOTAL SCORE ^**^	32.57 (15.4)	27.25 (12.0)	0.000°	31.87 (12.1)	28.85 (12.9)	0.005°
ITEM 3.10^**^	1.525 (0.96)	1.275 (0.78)	0.016°	1.550 (0.87)	1.359 (0.90)	0.130
ITEM 3.11^**^	0.500 (0.94)	0.500 (0.94)	1.00	0.650 (0.97)	0.447 (0.79)	0.153
ITEM 3.12^**^	1.400 (1.05)	1.025 (1.02)	0.009°	1.375 (1.03)	1.150 (1.02)	0.134
ITEM 3.13^**^	1.601 (1.17)	1.250 (1.03)	0.046	1.450 (1.19)	1.350 (1.18)	0.099

* , ANOVA for repeated measures;

** *, p-value. Wilcoxon test with MC method IC 99%; °, upper limit of the confidence interval of p < 0.050*.

#### Clinical Parameters

In the clinical variables, at the end of the active treatment we observed a significant decrease in the MDUPDRS Part III score from 32.6 to 27.3 *p* = 0.000 with significant decrease of ITEM 3 (*p* = 0.016). ITEM 3.12 from 1.40 (*p* = 0.009) and ITEM 3.13 (*p* = 0.046). No other significant difference at the end of active treatment was seen. In the Placebo treatment, we observed a significant decrease in MD UPDRS Part III score from 31.87 to 28.85 *p* = 0.005. No other significant difference at the end of Placebo treatment (see Table 3) was found.

### Effectiveness of Treatments and PD Impairment

**Figure 2:**
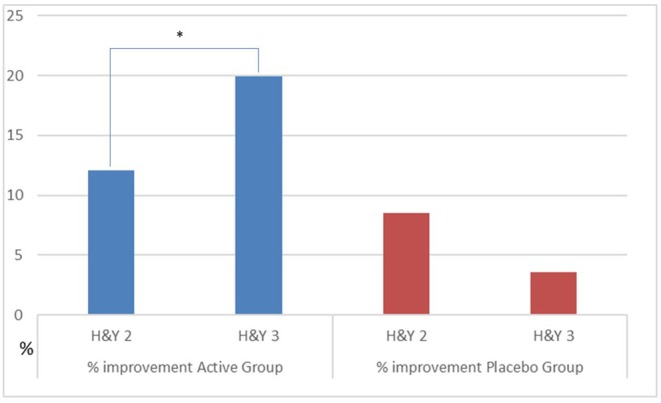
Percentage improvement of UPDRS III as H&Y factor in the group. Active vs. Placebo. *p ≤ 0.05.

**Figure 3:**
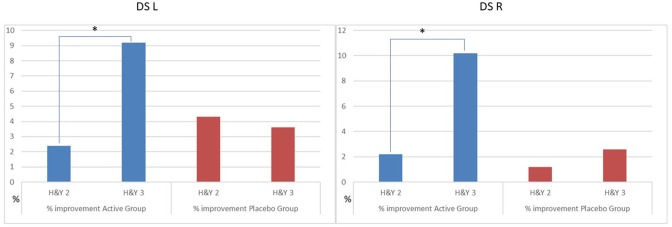
Percentage improvement of DS as H&Y factor in the group. Active vs. Placebo. DS, Double support; L, Left; R, right. *p ≤ 0.05.

We observed a different effect on Clinical and GA parameters by disease severity in the patients, during the active treatment. In the patients with H&Y = 2, the MDUPDRS III Total Score has an improvement of 12.1%. DST R and L a reduction of 2.4 and 2.2%, respectively. In the patients with H&Y = 3, MDUPDRS III Total Score had an improvement of 19.9% (see Figure 2). DS R and L a reduction of 9.2 and 10.2%, respectively (See Figure 3). Statistical differences between the two groups are reported in Table 4. Of note, a similar trend toward a more marked improvement in the more severely affected patients was also observed for mean velocity differences using H&Y factor. PD subjects with H&Y = 2 had a mean velocity improvement of 4.8%; the PD subjects with H&Y = 3 had an improvement of 15.7 %. The difference was however not statistically significant (*p* = 0.08). During the Placebo treatment, no differences with factor H&Y were observed.

**Table 4:** Significant differences in clinical scales and GA parameters in active group vs. placebo by H&Y factor using ANOVA with repeated measures.

	**UPDRS III—mean (±SD)**			**DST R—mean (±SD)**			**DST L—mean (±SD)**		
	**Before**	**After**	***p* value**	**Before**	**After**	***p* value**	**Before**	**After**	***p* value**
H&Y 2	23.8 (10.1)	20.9 (8.2)	0.050	12.5 (2.8)	12.2 (2.6)	0.048	13.2 (2.4)	12.9 (2.2)	0.038
H&Y 3	43.7 (14.0)	35 (12.8)		16.3 (4.2)	14.8 (3.8)		16.6 (4.1)	14.9 (3.8)	

## Discussion

The results of this study show that an intervention that mainly acts on the proprioceptive system improves motor performances in PD subjects. Several studies have suggested that peripheral afferent input may interfere with the processing of motor programs in the cortical motor areas (24). This hypothesis is also supported by electrophysiological findings showing that somatosensory evoked potentials (SEPs) and have an amplitude reduction of late component N30 that is related to cortical-subcortical loop that includes basal ganglia as well as supplementary motor area (SMA) (25). Moreover, contingent negative variation (CNV) of Event-Related Potentials (EP)—which reflects cognitive processes related to planning or anticipation of motor responses—is modified in amplitude and latency to suggest an abnormal processing of sensory inputs in PD patients (26, 27). Furthermore, the supplementary motor cortex is known to play an important role in connecting the sensory and the motor system (28) and it is also involved in the initiation of the anticipatory postural adjustments expressed in the DST (29). From a clinical point of view studies on rehabilitation trials based on exercises that stimulate the sensorimotor system such as the Blindfolder Balance Training (BBT) (1), recording TMS with and without BBT showed amelioration in Double Stance Support and modification of parameters of TMS (30) only in PD group with performed BBT. These findings confirm the important role of the indirect basal ganglia pathways on PD. Moreover, the most interesting results of our study not reported in a previous study (19) were that ameliorations were present in all the spatial-temporal parameters of GA, also those correlated to axial symptoms; the effect of the improvement related to severity of disease (29). In fact, both MDUPDRS III and Double Support ameliorated more in patients with H&Y: 3 than in those with H&Y: 2. Lastly, this is the first study in which an Equistasi® device was used in addition to pharmacological therapy without any rehabilitation training. Therefore, in terms of the severity of disease, it is tempting to hypothesize that the proprioceptive system is involved progressively more the more severe the disease is. In terms of spatiotemporal variables: in this study the increase in mean velocity did not reach the prior target of 10 cm/s as we recorded an increase of 5 cm/s, which was statistically significant only in active treatment. As reported in the literature and confirmed by our results, the mean velocity is the most frequently studied GA parameter and correlated to the severity of disease and cognitive impairment (31). In this perspective, we believe that a change in velocity might not be related to this kind of patients. Axial symptoms and gait bradykinesia are better studied by other specific variables of stride (8, 29, 32) as reported in our study. A possible pathophysiological explanation of the imperceptible action FVs through the CNS involves the phenomenon called stochastic resonance. This was observed in the neural tissue of sensory systems of several organisms in which an imperceptible stimulus, in a non-linear dynamic system like proprioceptive system, is added and amplified by noise (noise benefit) (33, 34). Then proprioceptive inputs, increasing cortical activation, induce an improvement of tendon muscle proprioceptive performance (35). Concerning the direct action of the Equistasi® device on extrapyramidal symptoms, in a previous study Equistasi®® was evaluated as an add-on to a physiotherapy program in 40 PD patients (12). This elegant study showed that the physiotherapy program for the balance of training in combination with FV delivered by a wearable proprioceptive stabilizer was superior to rehabilitation alone in improving the balance of patients (12). Walking problems and postural stability in PD are not well-controlled by pharmacological therapy and a surgical approach (36, 37) in moderate PD. Postural instability and falls lead to the use of advanced therapies for PD (38). That have poor results and are considered too invasive by patients-caregivers and physicians. The scarcity of treatments for these patients is real. Therefore, from a clinical point of view, our results open up an important speculative field with important practical implications. However, this study presents some limits: i.e., limited population number, as a large trial to would be needed to validate GA spatiotemporal variable as Velocity as well as more interesting study specific form of PD and Parkinsonism. In conclusion, this randomized controlled study indicates that the Equistasi® device is a useful aid in the treatment of middle-advanced PD in improving gait, posture and stability and could be used in addition to physiotherapy and overall, to pharmacological therapy.

## Data Availability

All datasets generated for this study are included in the manuscript/supplementary files.

## Ethics Statement

The studies involving human participants were reviewed and approved by IRCCS Fondazione Santa Lucia Roma Italy. The patients/participants provided their written informed consent to participate in this study.

## Author Contributions

AP: conceptualization. SP, MB, FS, LB, AG, ZS, DP, DC, NC, SB, and AM: methodology. ZS, AM, and MA: supervision. GA, GS, CT, DV, and AP: writing—review and editing.

### Conflict of Interest Statement

The authors declare that the research was conducted in the absence of any commercial or financial relationships that could be construed as a potential conflict of interest.
